# Bioactive Ingredients in the Mediterranean Diet for Hepatic Health: In Vitro Evaluation of Selected Extracts Against Non-Alcoholic Steatosis

**DOI:** 10.3390/molecules31193470

**Published:** 2026-09-29

**Authors:** Valentina Vassallo, Enrica Giustino, Rosario Finamore, Antonietta Stellavato, Nicola Alessio, Mariateresa Giuliano, Umberto Galderisi, Chiara Schiraldi

**Affiliations:** 1 Department of Experimental Medicine, Section of Biotechnology, Medical Histology and Molecular Biology, University of Campania “Luigi Vanvitelli”, 80138 Naples, Italy; v.vassallo@unilink.it (V.V.);; 2 Department of Life Sciences, Health and Health Professions, University of Study Link, 00165 Rome, Italy

**Keywords:** natural compounds, Mediterranean diet, HepG2 cells, NAFLD, PPARs, antioxidant activity

## Abstract

Natural products derived from the Mediterranean diet have attracted considerable interest as potential drug leads owing to their antioxidant and anti-inflammatory properties, as well as their beneficial effects on metabolic disorders such as non-alcoholic fatty liver disease (NAFLD). This study investigated the individual and combined effects of carvacrol, thymol, basil essential oil, and lycopene in an in vitro HepG2 model of hepatic steatosis. Their antioxidant activity was first confirmed by 2,2-diphenyl-1-picrylhydrazyl (DPPH) and 2,2′-azinobis-(3-ethylbenzothiazoline-6-sulfonic acid) (ABTS) radical-scavenging assays. Treatments significantly reduced intracellular lipid accumulation and lipid peroxidation, as demonstrated by Oil Red O staining, LipidSpot™ immunofluorescence, and the TBARS assay. In addition, the natural compounds restored lipid homeostasis by upregulating the β-oxidation- and lipolysis-related markers PPARα, CPT1α, and ATGL, while downregulating PPARγ, a lipogenic regulator, and the pro-inflammatory enzyme 5-lipoxygenase. The combined formulation consistently exhibited the strongest biological activity, showing enhanced efficacy in reducing lipid accumulation, oxidative stress, and oxidative DNA damage, as evidenced by decreased 8-oxo-7,8-dihydroguanine (8-oxo-dG) formation. Overall, these results demonstrate that Mediterranean-diet-derived bioactives, particularly when used in combination, exert multitarget antioxidant activity by reducing oxidative damage, restoring lipid homeostasis, and modulating inflammatory pathways, supporting their potential application in natural product-based strategies for the prevention and management of NAFLD.

## 1. Introduction

Nowadays, unhealthy lifestyles and poor dietary habits have increasingly affected human health from an early age, contributing to the rising global burden of metabolic disorders. Among these, non-alcoholic fatty liver disease (NAFLD) has emerged as one of the most prevalent chronic liver diseases worldwide. In this context, functional foods and their components have been identified as key agents in the prevention and treatment of liver disease in daily life [1]. However, lifestyle-based strategies, including calorie-controlled diets and regular physical exercise, are presently considered the first-line therapeutic option, particularly for children affected by hepatic steatosis. Conversely, studies investigating pharmacological treatments have produced inconsistent and often contradictory outcomes [2]. Concerning functional foods, they were introduced in Japan in 1991 and are characterized by health claims related to additional effects arising from the presence of naturally occurring components. These constituents interact, selectively, with one or more physiological functions of the human body (biomodulation), thereby leading to positive health benefits [3]. Moreover, they represent a valuable alternative to conventional dietary supplements, particularly for vulnerable populations such as children and elderly people, who may experience difficulties in swallowing solid dosage forms or are already subjected to poly-pharmacological regimens. In this context, food-based formulations such as soups and sauces offer a more accessible, acceptable, and palatable delivery system [4,5,6]. In fact, several components of functional foods have demonstrated the ability to prevent and treat NAFLD and liver fibrosis by different mechanisms, including anti-inflammatory activity, immune regulation, reduction in oxidative stress and oxidative damage, and modulation of lipid metabolism [7,8]. Interestingly, it has been described that specific Mediterranean herbs can naturally improve fat metabolism or prevent metabolic syndrome, type 2 diabetes mellitus and cardiovascular diseases [9]. In detail, the term Mediterranean diet refers to the traditional dietary patterns of related populations in the early 1960s, which were associated with high life expectancy and a low incidence of cardiovascular and other age-related chronic diseases [10]. This eating pattern, which is predominantly plant-based, is characterized by a high intake of vegetables, legumes, whole grains, fruits, nuts, and seeds; extra virgin olive oil as the primary source of fat; limited consumption of red meat; and moderate intake of fish, dairy products, and wine [11].

Given its low content of saturated fats (~10% of total energy) and its richness in bioactive compounds, including vitamins, carotenoids, unsaturated fatty acids, and plant-derived phenolic compounds with antioxidant and anti-inflammatory properties, the Mediterranean diet represents a valuable source of natural compounds capable of modulating metabolic and oxidant-related pathways. These bioactives may contribute to maintaining redox homeostasis by limiting reactive oxygen species (ROS)-mediated cellular damage, thereby supporting improved insulin sensitivity and metabolic health [9,12]. In this context, polyphenols and terpenoids play a predominant role; notably, aromatic herbs such as basil, thyme, and oregano, widely used in the dietary regimen of Mediterranean countries, appear to contribute significantly to these beneficial effects, being rich in bioactive molecules such as eugenol, thymol, and carvacrol. These compounds have been associated with the promotion of well-being and, when consumed at specific dosages, have also been evaluated by the European Food Safety Authority (EFSA) for their potential health claims [13]. Importantly, their biological activity is strongly influenced by concentration, as several plant-derived compounds may exert antioxidant or pro-oxidant effects depending on the cellular context and dose. Among the foods contributing to this bioactive profile, tomatoes, key components of the Mediterranean diet, are a rich source of health-promoting compounds and have been associated with protective effects against diabetes, hypertension, hepatic, and cardiovascular disorders. These effects are mainly attributed to carotenoids (lycopene, phytofluene), vitamins (C, E, B12), steroidal glycoalkaloids (α-tomatine, tomatidine), and various phenolic acids and flavonoids (e.g., ferulic, caffeic, quercetin, kaempferol, delphinidin, resveratrol). Although thermal processing may reduce some thermolabile compounds, carotenoids such as lycopene and several polyphenols remain stable or may even become more bioavailable due to matrix softening [14]. Tomato-derived carotenoids, especially lycopene, exert strong antioxidant effects by protecting lipids, proteins, and DNA from ROS-induced oxidative damage [15,16]. Consistently, recent studies in NAFLD rat models have shown that tomato juice supplementation improves high-fat diet-induced metabolic alterations, further supporting the potential hepatoprotective role of tomato-derived bioactive compounds [17,18]. Tomatoes are commonly consumed with basil and oregano, herbs rich in phenolic compounds that further enhance the antioxidant potential of the Mediterranean diet. Preclinical studies indicate that basil essential oil restores hepatic antioxidant levels, reduces lipid peroxidation, and counteracts oxidative imbalance in rats [19]. Carvacrol and thymol, phenolic monoterpenes present in oregano and thyme, have demonstrated hepatoprotective effects in models of hepatocellular carcinoma [20]. Eugenol (present in basil), a component of clove essential oil (EO), is also well documented for its antioxidant and anti-inflammatory properties [21].

However, the pathogenesis of NAFLD and its progression to non-alcoholic steatohepatitis (NASH), which can further advance to cirrhosis and hepatocellular carcinoma through a complex biochemical mechanism, has not been fully elucidated. The scientific community agrees that hepatic fat accumulation and increased oxidative stress are hallmarks of NAFLD pathogenesis, driving lipid peroxidation, inflammation, mitochondrial dysfunction, and disease progression, together with obesity, insulin resistance, and chronic inflammation [22,23]. Hepatic damage often originates from abnormal visceral fat accumulation, a condition that is particularly detrimental when it begins in childhood [24,25]. Peroxisome proliferator-activated receptors (PPARs) play a key role in regulating fatty acid metabolism and storage [26], and emerging evidence suggests that lycopene, a fat-soluble compound found in tomatoes and other fruits and vegetables, may exert protective effects in NAFLD partly through modulation of PPAR activity, in addition to inactivating ROS and scavenging free radicals, thereby contributing to the restoration of redox balance in hepatic cells [27]. Finally, a recent in vivo study showed that dietary supplementation with tomato juice enriched in lycopene, eugenol, carvacrol, and thymol not only improves glucose and lipid metabolism in obese children with NAFLD but also improves antioxidant defenses, reduces oxidative stress-related alterations, and decreases inflammatory markers [2].

Building on previous in vivo studies [2,28], although preclinical and clinical studies have demonstrated antioxidant, redox-modulating, and hepatoprotective effects of basil, carvacrol, thymol, eugenol, and lycopene individually, their combined biological activity has never been systematically investigated within a single experimental setting [29,30,31,32,33,34].

Moreover, recent evidence has shown that carvacrol, thymol, and lycopene can function as potent senotherapeutics in mesenchymal stromal cells, selectively eliminating senescent cells through controlled pro-oxidant mechanisms. This highlights the versatile bioactivity of these terpenes, acting as either protective or cytotoxic agents depending on the dosage and cellular context [35].

For this reason, this study aimed to investigate the key biochemical markers modulated by carvacrol, thymol, basil essential oil, and lycopene, compounds commonly found in the Mediterranean diet, both individually and in combination, using an in vitro NAFLD model based on HepG2 cells. Understanding the contribution of each component, as well as their combined effects, may guide the development of targeted food supplements or functional foods aimed at restoring redox homeostasis while modulating oxidative stress, lipid dysregulation, and inflammatory pathways involved in NAFLD progression. Furthermore, assessing whether combinations of these compounds at lower doses can maintain or enhance bioactivity compared with higher doses of the individual agents may facilitate the development of safer and more effective dietary strategies for NAFLD prevention and management.

## 2. Results

### 2.1. Analytical Characterization and Antioxidant Evaluation

UPLC analysis confirmed the high purity of lycopene, carvacrol, and thymol, each showing a single peak accounting for ~25% (*w*/*v*). Basil essential oil was mainly characterized by eugenol (23%), with trace amounts (<1%) of carvacrol and thymol. Based on the concentrations determined by UPLC and calibration with appropriate standards (Supplementary Figure S1), extracts/essential oils and lycopene were subsequently tested for their antioxidant activity, alone and in combination. Increasing concentrations of carvacrol, thymol, basil essential oil, and lycopene (0.003–7 μM) were evaluated for their radical scavenging activity against DPPH and ABTS, in comparison with ascorbic acid. As shown in Figure 1, all compounds exhibited dose-dependent antioxidant activity, although to different extents. Basil essential oil showed the highest activity, reaching 85 ± 3% inhibition at 0.8 μM (Figure 1A), compared to 66 ± 7%, 68 ± 2%, and 57 ± 3% inhibition for thymol, carvacrol, and lycopene, respectively, at the same concentration. However, all essential oils achieved approximately 90% inhibition at the highest concentration tested. A similar trend was observed for ABTS radical scavenging activity, which was comparable to that of ascorbic acid (Figure 1B).

### 2.2. Biocompatibility Evaluation by MTT Assay

To evaluate the potential use of essential oils and lycopene as nutraceutical agents against lipid accumulation in steatotic-like HepG2 cells, their biocompatibility was assessed. Cells were treated with increasing concentrations of natural compounds (0.1–2.5 μM) for 24 and 48 h. As shown in Figure 2, EOs and lycopene, tested alone or in combination, were fully biocompatible under all experimental conditions.

### 2.3. Determination of Total Lipid Accumulation by Oil Red O Staining

Following steatosis induction, intracellular lipid accumulation was assessed by Oil Red O staining. As shown in Figure 3, FFA-treated cells (Figure 3B) exhibited a marked increase in lipid droplets compared to control cells (Figure 3A), confirming the establishment of a steatotic phenotype. Treatment with nutraceuticals after FFA exposure led to a clear reduction in lipid accumulation (Figure 3C–G). These observations were supported by spectrophotometric quantification of Oil Red O extracted with isopropanol. As reported in Figure 3H, absorbance at 510 nm was significantly reduced in all treated samples compared to FFA-treated cells, showing a similar trend across conditions. Notably, the mixture achieved a slightly but significantly greater reduction in lipid content (≤0.0001).

### 2.4. Immunofluorescence Evaluation of Lipid Accumulation

Immunofluorescence analysis corroborated the observations obtained by Oil Red O staining. As shown in Figure 4, FFA-treated cells (Figure 4B) exhibited a higher green fluorescence signal compared to control cells (Figure 4A), indicating increased lipid accumulation. Treatment with natural antioxidant bioactives, either alone or in combination, reduced intracellular lipid content compared to FFA-treated cells, with more pronounced effects observed in thymol (Figure 4D), basil essential oil (Figure 4E), and the mixture (Figure 4G) samples. Quantitative analysis of lipid droplet accumulation (Figure 4H) confirmed a significant reduction in fluorescence intensity following thymol, basil essential oil, and mixture treatments, with the latter showing the greatest effect (1.6-fold decrease vs. FFAs).

### 2.5. Gene Expression Evaluation by qRT-PCR

As shown in Figure 5, FFA treatment efficiently induced a steatotic phenotype, significantly increasing PPARγ and 5-LOX expression (Figure 5B and Figure 5E, respectively), while reducing PPARα, CPT1α, and ATGL expression compared to control cells (Figure 5A, Figure 5C, and Figure 5D, respectively). Treatment with EOs and lycopene counteracted lipid accumulation-related alterations. All treatments significantly downregulated PPARγ expression (Figure 5B), with fold reductions of 4.42, 3.57, and 3.48 for lycopene, basil essential oil, and the mixture, respectively, whereas carvacrol showed no significant effect. Conversely, all compounds enhanced β-oxidation, as indicated by the upregulation of both PPARα and CPT1α (Figure 5A,C). Basil essential oil induced the strongest increase in PPARα expression (7.6-fold), while the mixture was the most effective in upregulating CPT1α (13.5-fold). Lipolytic activity was also improved, with increased ATGL expression observed in all treated groups (Figure 5D), ranging from a 5.5-fold increase with the mixture to a 7.6-fold increase with carvacrol. Finally, 5-LOX expression was significantly downregulated by carvacrol, thymol, and the mixture, with a decreasing trend also observed for lycopene, whereas basil essential oil showed no effect. The mixture exerted the strongest inhibitory effect, reducing 5-LOX expression by approximately 2.27-fold.

### 2.6. Protein Expression Evaluation by Western Blot

Western blot analyses were performed on protein lysates obtained after 48 h of treatment with EOs and lycopene. The results for 5-LOX, PPARα, and PPARγ were consistent with those obtained by qRT-PCR. As shown in Figure 6, 5-LOX protein expression was significantly upregulated in FFA-treated cells compared with control (*p* < 0.05), confirming the induction of a steatotic-like phenotype, whereas treatment with all EOs and lycopene significantly reduced its expression. In particular, lycopene and the mixture induced a significant decrease (*p* < 0.05) of approximately 1.7-fold and 1.9-fold, respectively, compared with FFA-treated cells. PPARα expression, which was inhibited by FFAs, was significantly increased following treatment with nutraceutical bioactives, with the mixture showing the strongest effect (4-fold increase vs. FFAs; Figure 6). Conversely, PPARγ was upregulated by FFAs compared with control cells (*p* < 0.05) but was downregulated after EO and lycopene treatments. Again, the mixture was the most effective treatment (*p* < 0.05), reducing PPARγ protein levels by 3.2-fold with respect to FFA-treated samples.

### 2.7. Lipid Peroxidation Assay (TBARS)

Results related to the protective effects of EOs and lycopene (Figure 7A) showed a significant increase in lipid peroxidation in cells treated with H_2_O_2_ and FFAs + H_2_O_2_ compared with untreated controls, confirming the induction of oxidative stress. Treatment with carvacrol, basil essential oil, and thymol induced a modest reduction in lipid peroxidation compared with FFAs + H_2_O_2_. Notably, lycopene significantly decreased lipid peroxidation, indicating a marked antioxidant effect (*p* < 0.05 vs. FFAs + H_2_O_2_). The combined formulation also significantly reduced lipid peroxidation compared with FFAs + H_2_O_2_ (*p* < 0.05), indicating an enhanced protective effect compared with the pathological condition.

The ability of natural compounds to restore lipid peroxidation levels (repair function, Figure 7B) was also evaluated. All treatments significantly reduced lipid peroxidation compared with FFAs + H_2_O_2_, with the mixture showing the strongest effect, leading to an approximately 18-fold decrease and representing the most effective condition in counteracting oxidative damage. Overall, these results highlight the protective and reparative effects of the combined formulation against oxidative stress-induced lipid peroxidation, with a more pronounced effect observed in the repair setting.

### 2.8. 8-Oxoguanina Detection (Anti-8-oxo-dG) Related to Oxidative DNA Damage

Among the detrimental effects of H_2_O_2_, oxidative DNA damage includes the formation of 8-oxo-7,8-dihydroguanine (8-oxo-dG). Accordingly, we quantified the percentage of 8-oxo-dG-positive HepG2 cells following H_2_O_2_ exposure in the presence or absence of natural compounds (Figure 8). Immunocytochemistry analysis showed a significant reduction (*p* < 0.05) in the percentage of 8-oxo-dG-positive cells after treatment with basil essential oil, lycopene, and the mixture compared with H_2_O_2_ + FFA-treated cells (Figure 8J). Specifically, basil essential oil, lycopene, and the mixture reduced 8-oxo-dG-positive cells by approximately 1.2-, 1.6-, and 5-fold, respectively. The effects of carvacrol and thymol were less pronounced.

## 3. Discussion

NAFLD is a prevalent chronic liver disease characterized by excessive lipid accumulation, hepatic steatosis, and impaired redox homeostasis resulting from an imbalance between reactive oxygen species production and antioxidant defenses. Oxidative stress represents a key driver of disease progression, contributing to lipid peroxidation, inflammation, and hepatocellular dysfunction. Therefore, strategies aimed at restoring redox homeostasis while reducing lipid accumulation may represent promising approaches for NAFLD prevention and management [36]. In vitro models based on HepG2 or L02 cell lines are widely used to investigate NAFLD-related molecular mechanisms, with oleic acid (OA) and palmitic acid (PA) commonly employed to induce intracellular lipid accumulation [37,38,39]. In this experimental set-up, we investigated the molecular mechanisms underlying the effects of carvacrol, eugenol, thymol, and lycopene on lipid metabolism, oxidative stress, and inflammation, both individually and in combination, using a well-established HepG2-based in vitro NAFLD model [40].

Antioxidant properties have been reported for all the phytochemicals investigated in the present study, although the strength of the available evidence is not uniform across the different molecules [41,42]. Basil essential oil carvacrol, thymol, and lycopene have been investigated to different extents, providing evidence of their antioxidant and metabolic effects. Basil essential oil has been widely explored in in vitro and animal studies, which consistently demonstrate its antioxidant potential; however, clinical investigations on basil-based commercial supplements remain limited, highlighting the need for further validation in human studies [43]. However, it must be specified that basil essential oils are complex mixtures whose chemical composition can vary considerably according to genotype, chemotype, geographical origin, and cultivation conditions. In a study of 38 *O. basilicum* accessions, Zheljazkov et al. identified a linalool-eugenol chemotype in which eugenol accounted for 5–29% of the essential oil [44]. Consistently, a more recent analysis of *O. basilicum* essential oil obtained from plants cultivated in southern Italy reported eugenol as one of the major constituents, accounting for 20.4% of the oil [45]. The eugenol content measured in the basil essential oil used in the present study (approximately 23%) therefore falls within the compositional variability previously reported for *O. basilicum* essential oils. Importantly, the biological activity observed in the present study should be considered in the context of the complex chemical composition deriving from any extraction of the basil leaves and should not be attributed exclusively to eugenol. However, in all the scientific reports cited, eugenol was either the major component or among the 2–3 most represented chemical species. Similarly, carvacrol and thymol, two phenolic monoterpenes commonly present in aromatic plants, have been shown in experimental models to reduce oxidative damage and modulate antioxidant defense systems [41,43]. Despite these encouraging preclinical data, clinical studies specifically evaluating supplementation with basil-, carvacrol-, or thymol-based formulations remain limited, and the available evidence often derives from their use in combination with other bioactive compounds in dietary formulations. In contrast, lycopene is supported by the most extensive experimental and clinical evidence [46]. Several randomized controlled trials and meta-analyses conducted in healthy subjects and specific populations have reported improvements in antioxidant status, together with reductions in biomarkers of oxidative damage, including lipid oxidation and oxidative DNA damage, following lycopene supplementation [47]. Collectively, these data suggest that the biological activity of Mediterranean-derived compounds extends beyond direct radical scavenging, involving the modulation of oxidative stress-related pathways and the preservation of cellular homeostasis. In the present study, we evaluated the combined effects of these four Mediterranean-derived phytochemicals in a single experimental model to investigate whether combining compounds with complementary mechanisms could enhance their biological activity. This approach is consistent with the Mediterranean dietary pattern, in which multiple bioactive molecules are naturally consumed together and may contribute to complementary or additive biological effects [10,12,42].

Moreover, it should be noted that many polyphenols have been reported to exert cytotoxic effects on cancer cells, and the cytotoxic activity of EOs on HepG2 cells has also been described. In particular, phytochemicals such as eugenol and carvacrol have been shown to reduce HepG2 cell viability in a dose-dependent manner; for instance, eugenol exhibited an IC_50_ of approximately 189 µg/mL in HepG2 cells, while essential oils rich in eugenol have been reported to significantly decrease HepG2 cell survival at concentrations as low as ~10 µg/mL, and carvacrol has been shown to induce growth inhibition and apoptosis in the same cell line [48,49,50]. Therefore, sub-cytotoxic concentrations were selected here to preserve HepG2 cell physiology while enabling a reliable in vitro model of NAFLD. This choice was also guided by the well-established dose-dependent behavior of phenolic compounds, which may exert either antioxidant or pro-oxidant effects depending on their concentration and cellular context [51]. At low concentrations, these molecules generally act through free-radical scavenging mechanisms, contributing to the prevention of lipid oxidation. Accordingly, the DPPH assay was performed to evaluate the radical-scavenging activity of the EOs and lycopene [37,52,53]. In our experimental set-up, antioxidant activity was already detectable at low micromolar concentrations (Figure 1). Consistent with these results, the tested compounds subsequently reduced both lipid peroxidation and oxidative DNA damage.

These findings are noteworthy, as such amounts are compatible with levels of intake achievable through a functional food, particularly within the context of the Mediterranean diet. Indeed, a single basil leaf has been reported to contain, on average, approximately 136 µg of eugenol [37,52,53]. Our results demonstrated that treatment with lycopene and the tested EOs reduced intracellular lipid droplet accumulation, with the combined formulation showing the strongest effect, particularly in immunofluorescence analyses. Since excessive lipid accumulation promotes organelle dysfunction, ROS generation, lipid peroxidation, and disease progression, it represents a key therapeutic target in NAFLD [23,54]. Lipid droplet dynamics are regulated by PPARα and PPARγ, central modulators of hepatic lipid homeostasis: PPARα promotes fatty acid β-oxidation, whereas PPARγ enhances lipid storage, contributing to insulin resistance and steatosis [55,56]. Accordingly, our data indicate that the investigated molecules modulate PPARα and PPARγ in opposite directions, suggesting a protective effect through enhanced lipid degradation and restoration of lipid homeostasis. This metabolic reprogramming may limit ROS generation and oxidative damage, thereby attenuating oxidative stress and inflammation, two key drivers of NAFLD progression [54,55,57,58,59]. Lipid dysregulation and inflammation are also closely interconnected through the arachidonic acid/5-lipoxygenase (5-LOX) pathway, which generates pro-inflammatory leukotrienes involved in NAFLD pathogenesis. Moreover, 5-LOX activation contributes to oxidative stress, as lipid hydroperoxides produced during oxidative damage further promote leukotriene biosynthesis, establishing a feed-forward loop between ROS production and inflammation [60]. Increased 5-LOX activity correlates with disease severity, while its inhibition improves hepatic outcomes, supporting its relevance as a therapeutic target [61]. In line with these observations, the tested EOs and lycopene reduced 5-LOX expression, with the combined formulation showing a stronger effect than the individual treatments. An important aspect of this work is that the concentrations of carvacrol, thymol, and lycopene used (0.5–2 μM) are far below those typically required to exert pro-oxidant or cytotoxic effects. Indeed, our group recently demonstrated that at higher dosages, these same compounds act as senotherapeutics by purposely increasing ROS levels to trigger apoptosis in senescent mesenchymal stromal cells. This is consistent with the reported hormetic behavior of several dietary phytochemicals, whose biological effects may vary depending on concentration and cellular context. At the low micromolar doses applied here, they predominantly exerted antioxidant and cytoprotective effects, reducing lipid-induced oxidative stress in HepG2 cells, whereas they act as pro-oxidant cell-clearing agents at much higher concentrations in a senescent environment [35]. These observations reinforce the concept that the biological activity of Mediterranean phytochemicals is strongly dose- and context-dependent, with low concentrations primarily promoting cytoprotection and redox homeostasis rather than pro-oxidant effects. From a nutritional perspective, the concentrations used in vitro should not be directly equated with dietary exposure, as absorption, metabolism, bioavailability, and tissue distribution substantially influence the concentrations reached in vivo. In the Italian population, the average dietary intake of lycopene has been estimated at approximately 7.4 mg/day, mainly derived from tomatoes and tomato-based products [62]. In contrast, reliable estimates of habitual dietary exposure to carvacrol and thymol in humans remain limited, although human intervention studies have demonstrated systemic exposure to thymol metabolites following oral intake [63,64]. Therefore, the concentrations investigated in the present in vitro model should not be interpreted as directly achievable dietary concentrations or as evidence supporting supplementation at pharmacological levels. Overall, these results suggest that the protective effects of Mediterranean-diet-derived molecules involve multiple interconnected mechanisms, with redox regulation emerging as a central component of their biological action. The reduction in 8-oxo-dG-positive cells further supports the antioxidant and cytoprotective properties of the tested phytochemicals, indicating decreased oxidative DNA damage associated with reduced ROS generation and lipid peroxidation, potentially linked to 5-LOX pathway modulation [65,66]. From a translational perspective, the present study supports the potential use of these compounds as functional ingredients for the management of NAFLD-associated alterations. The enhanced activity observed with the combined formulation suggests that multi-component approaches may provide added value compared with single compounds, potentially through complementary mechanisms. However, dietary intake of key molecules, including lycopene, is often suboptimal, supporting the development of functional foods or nutraceutical strategies to achieve biologically relevant doses [67,68,69]. In conclusion, Mediterranean-diet-derived phytochemicals represent promising natural products capable of reducing oxidative damage, restoring lipid homeostasis, and modulating inflammatory pathways involved in NAFLD. Their combined use may provide complementary effects compared with individual agents, supporting the development of functional food strategies. Further in vivo and clinical studies are required to validate these findings, clarify the potential interactions among the compounds, and define effective intake levels.

## 4. Materials and Methods

### 4.1. Natural Extracts

The natural extracts and bioactive compounds used in this study were carvacrol, thymol, lycopene and basil essential oil obtained by steam distillation and characterized by a high eugenol content. These compounds and the basil essential oil were previously obtained by a food company and used for the preparation of enriched tomato-based puree within the PON03PE_000060_3 project in collaboration with the University of Naples Federico II (Prof. Greco, School of Medicine) [34].

### 4.2. Analytical Method (UPLC) for the Determination of Carvacrol, Basil Essential Oil and Thymol

The analyses of the extracts were conducted using a UPLC Ultimate 3000+ system (Thermo Fisher, Milan, Italy), connected to the software Chromeleon (Thermo Fisher, Milan, Italy). All samples were dissolved in methanol at a concentration of 1 mg/mL and loaded onto an RP column (EC250/4.6 NUCLEODUR 100-5 C18ec, Macherey-Nagel, Del Chimica, Naples, Italy). The standards were dissolved in methanol at concentrations ranging from 5.0 to 0.0097 mg/mL. The buffers used were A: 0.1% trifluoroacetic acid in water; B: 0.1% trifluoroacetic acid in acetonitrile. The samples were eluted by applying a step gradient of buffer B: 0.0 min %B = 5.0; 5.0 min %B = 5.0; 35.0 min %B = 95.0; 40.0 min %B = 95.0; 45.0 min %B = 5.0; 50.0 min %B = 5.0, at a flow rate of 1 mL/min at 25 °C. The chromatographic profiles were registered by detecting the signal at 280 nm. Eugenol was quantified using a commercial eugenol standard, and its concentration in the basil essential oil was approximately 23% (*w*/*w*).

### 4.3. Evaluation of In Vitro Antioxidant Activity

To evaluate carvacrol, thymol, basil essential oil, and lycopene antioxidant activity, two in vitro colorimetric assays were performed: the 2,2-difenil-1-picrilhydrazyl (DPPH) assay and the 2,2-Azino-bis (3-ethylbenzothiazoline-6-sulfonic acid) (ABTS) assay [70]. The DPPH assay was performed as reported by Brand–Williams with some modifications [71]. Briefly, 0.1 mL of each sample, tested at different concentrations (0.003–7 µM), was incubated with 0.9 mL of methanolic DPPH solution (0.1 mM). After vortexing, the mixture was allowed to react for 30 min at room temperature, in the dark. At the end of incubation, the absorbance was measured spectrophotometrically at 517 nm. Ascorbic acid was used as the standard. Results are reported as percentage of DPPH inhibition with respect to control (i.e., absorbance of the sample in the absence of any molecule), following the equation

$$Inhibition\left(\%\right)=\left(1-\frac{{Abs}_{517sample}}{{Abs}_{517control}}\right)\times 100$$

As for the ABTS assay, all the molecules tested at different concentrations (0.003–7 µM) and were allowed to react with ABTS^•+^ for 7 min in the dark, and the absorbance was measured at 734 nm. Ascorbic acid was used as a standard to obtain a calibration curve. Results are reported as percentage of ABTS inhibition with respect to control (i.e., absorbance of the sample in the absence of any molecule) following the equation

$$Inhibition\left(\%\right)=\left(1-\frac{{Abs}_{734sample}}{{Abs}_{734control}}\right)\times 100$$

All experiments were performed in triplicate.

### 4.4. Cell Culture and Biocompatibility Evaluation by MTT Assay

Human hepatic cancer cells (HepG2) were purchased from ATCC (Manassas, VA, USA) and cultured in Dulbecco’s modified Eagle’s medium (DMEM) (GIBCO, BRL Life Technologies, Grand Island, NY, USA), supplemented with 10% fetal bovine serum, 2 mM L-glutamine, and antibiotics at 37 °C in a 5% CO_2_ humidified atmosphere. The biocompatibility of carvacrol, thymol, basil essential oil, lycopene and their mixture was evaluated by the MTT assay [72]. Briefly, HepG2 cells were seeded in a 96-well plate at a density of 5 × 10^3^ cell/well and incubated with increasing concentrations of each molecule (0.1–2.5 μM) for 24 and 48 h. At the end of incubation, cell viability was assessed spectrophotometrically by reading the absorbance at 560 nm (Beckman DU 640 spectrometer (Beckman, Milan, Italy)). Cell viability was expressed as the percentage of viable cells in the presence of the extract compared to the controls, represented by untreated cells and cells incubated with identical volumes of DMSO. The experiments were performed in triplicate, and the results are shown as mean ± SD.

### 4.5. In Vitro Non Alcoholic Hepatic Steatosis Induction in HepG2 Cells

To efficiently induce lipid accumulation in HepG2, mimicking an in vitro steatosis model, cells were seeded in 6-well plates at 1.5 × 10^5^ cell/well concentration and incubated with 6 mM of a mixture of linoleic (a poly-unsaturated 18:2, ω-6 fatty acid) and oleic (monounsaturated 18:1, ω-9 fatty acid) acid, ratio 1:1 *v*/*v* (L9655-Sigma Aldrich, Milan, Italy), for 24 h [40]. After the incubation, cells were washed in Phosphate-Buffered Saline (PBS) and treated for 24 or 48 h with 2 µM. Additionally, a potential formulation was obtained by mixing 0.5 µM of the single substances to reach a 2 µM final concentration.

### 4.6. Determination of Total Lipid Accumulation by Oil Red O Staining

The intracellular neutral lipid accumulation was evaluated by Oil Red O staining according to Stellavato and collaborators [40]. Briefly, cells were seeded in 24-well plates at a density of 1 × 10^5^ then treated with FFAs and EOs as previously reported. After the treatment, cells were washed with PBS and fixed in 4% Paraformaldehyde for 15 min at room temperature in the dark. After fixation, cells were washed with PBS, then with 60% isopropanol, and allowed to dry. Untreated cells and cells treated with EOs and lycopene were incubated with Oil Red O working solution (5.2 mM in isopropanol) for 20 min in the dark. Finally, cells were extensively washed with distilled water, and optical microscopy images were acquired (Leica Microsystems, Wetzlar, Germany) at 20× magnification. Intracellular Oil Red O quantification was obtained by adding pure isopropanol and by measuring the absorbance at 510 nm through a Beckman DU 640 spectrometer (Beckman, Milan, Italy). The experiments were performed in triplicate and the results are shown as mean ± SD.

### 4.7. Immunofluorescence Evaluation of Lipid Accumulation

Immunofluorescence analyses were performed according to the method previously described [73] with appropriate modifications. Specifically, HepG2 cells were fixed with 4% paraformaldehyde for 10 min and then permeabilized with a solution of 0.3% triton X-100, 1% Bovine Serum Albumin and 5% Fetal Bovine Serum in PBS for 1 h at room temperature in the dark. After that, LipidSpot™ (Biotium, Fremont, CA, USA) was diluted 1:200 in PBS, and cells were incubated with it at room temperature for 10 min protected from light. TRITC-conjugated fluorescent phalloidin (50 µg/mL) was employed here to stain actin filaments, and slides were covered using mounting medium with DAPI-aqueous fluoroshield. Finally, cells were mounted for observation. Fluorescence images were acquired using an Axiovert 200 fluorescence microscope (Carl Zeiss Microscopy GmbH, Jena, Germany) at 40× magnification and analyzed with AxioVision 4.8.2 software.

### 4.8. RNA Extraction and Gene Expression Evaluation by qRT-PCR

The effects of EOs and lycopene after 24 h incubation in steatotic HepG2 cells on the expression of lipid metabolism-related genes were analyzed by quantitative real-time polymerase chain reaction (qRT-PCR), as previously reported [74]. Genes were selected based on their roles in lipid metabolism: (i) Peroxisome Proliferator-Activated Receptor Alpha (PPARα), which promotes fatty acid catabolism via mitochondrial and peroxisomal β-oxidation; (ii) Peroxisome Proliferator-Activated Receptor Gamma (PPARγ), which drives lipid storage and lipogenesis, contributing to hepatic steatosis when overexpressed; (iii) Carnitine Palmitoyltransferase 1A (CPT1α), the rate-limiting enzyme for mitochondrial fatty acid import; (iv) Adipose Triglyceride Lipase (ATGL), which hydrolyzes triglycerides to release free fatty acids; and (v) 5-Lipoxygenase (5-LOX), which converts arachidonic acid into pro-inflammatory leukotrienes. qRT-PCR was performed using iQ™ SYBR^®^ Green Supermix (Bio-Rad Laboratories, Milan, Italy), with glyceraldehyde-3-phosphate dehydrogenase (GAPDH) as the housekeeping gene. Relative gene expression was calculated using the 2^−ΔΔCt^ method [75]. Primer sequences used for qRT-PCR are reported in Table 1. Experiments were performed in triplicate, and results are presented as mean ± SD.

### 4.9. Protein Extraction and Relative Evaluation by Western Blotting

After 48 h of treatment with EOs and lycopene, cells were harvested using trypsin/EDTA (0.2 mg/mL) and lysed in RIPA buffer (1×; Cell Signaling Technology, Danvers, MA, USA). Protein concentration was determined using the Bradford assay (Bio-Rad Laboratories, Milan, Italy). Western blotting was performed as previously described [76]. Briefly, 20 μg of total protein was separated by 10% SDS-PAGE and transferred onto nitrocellulose membranes (GE Healthcare, Amersham, UK) at 100 V for 1 h. Membranes were blocked with 5% non-fat milk in Tris-buffered saline containing 0.05% Tween-20 (TBST) (Sigma-Aldrich, St. Louis, MO, USA) for 20 min and incubated overnight at 4 °C with primary antibodies against PPARα, PPARγ, and 5-LOX (Santa Cruz Biotechnology, Dallas, TX, USA; 1:250 dilution). After washing, membranes were incubated with secondary antibodies (Santa Cruz Biotechnology, Dallas, TX, USA; 1:250 dilution; 1:5000 dilution) for 1 h at room temperature. β-Actin (1:1000 dilution) was used as a loading control. Protein bands were visualized using an enhanced chemiluminescence (ECL) detection system (Merck Millipore, Burlington, MA, USA), and densitometric analysis was performed using ImageJ software 1.50 (Windows version). Experiments were performed in triplicate, and results are presented as mean ± SD.

### 4.10. Lipid Peroxidation Assay (TBARS)

Lipid peroxidation was assessed by the thiobarbituric acid reactive substances (TBARS) assay. In this experimental set-up, EOs, lycopene, and their mixture were evaluated as both protective and reparative agents. For the protection protocol, steatotic cells were pre-treated for 24 h with natural compounds and then exposed to 50 μM H_2_O_2_ for 30 min. For the repair protocol, steatotic cells were first exposed to 50 μM H_2_O_2_ for 30 min and subsequently treated with natural compounds for 24 h. After treatments, cells were lysed in RIPA buffer (1×; Cell Signaling Technology, Danvers, MA, USA), and protein concentration was determined using the Bradford assay (Bio-Rad Laboratories, Milan, Italy). Lipid peroxidation was quantified by the TBARS assay as previously described [40]. Results are expressed as percentage reduction relative to FFAs + H_2_O_2_-treated cells. Experiments were performed in triplicate, and results are presented as mean ± SD.

### 4.11. Oxoguanin Detection (Anti-8-oxo-dG) Related to Oxidative DNA Damage

To further assess oxidative DNA damage in H_2_O_2_-treated cells, 8-oxo-2′-deoxyguanosine (8-oxo-dG) was detected by immunofluorescence, according to the method described by Stuart and collaborators [77], using an anti-8-oxo-dG primary antibody (clone 2E2, Euroclone S.p.A., Pero (MI), Italy). Nuclei were counterstained with Hoechst 33342. Samples were observed using an Axiovert 200 fluorescence microscope (Carl Zeiss Microscopy GmbH, Jena, Germany), and images were analyzed with AxioVision software (version 4.8.2).

### 4.12. Statistical Analysis

Results are expressed as mean ± standard deviation (SD). Statistical significance was assessed using a two-tailed *t*-test (*p* < 0.05) or one-way analysis of variance (ANOVA) followed by Bonferroni’s post hoc test, as appropriate. All analyses were performed using GraphPad Prism for Windows (version 10.01). In all cases, the specific statistical test applied is indicated in the corresponding figure legends.

## 5. Conclusions

In conclusion, Mediterranean-diet-derived phytochemicals represent promising natural compounds capable of attenuating oxidative damage, modulating lipid homeostasis, and influencing inflammatory pathways involved in NAFLD-related cellular alterations. Under the experimental conditions investigated, their combined treatment resulted in more pronounced effects on several parameters compared with individual treatments, although further studies are required to clarify the nature of the interactions among the compounds. These findings provide mechanistic evidence supporting the potential biological activity of Mediterranean-diet-derived phytochemicals in an in vitro model of NAFLD and highlight redox modulation as a relevant component of their cellular effects. Further in vivo and clinical studies are needed to determine whether these observations can be reproduced in more complex biological systems and to establish their potential relevance to dietary or nutraceutical applications.

## Figures and Tables

**Figure 1:**
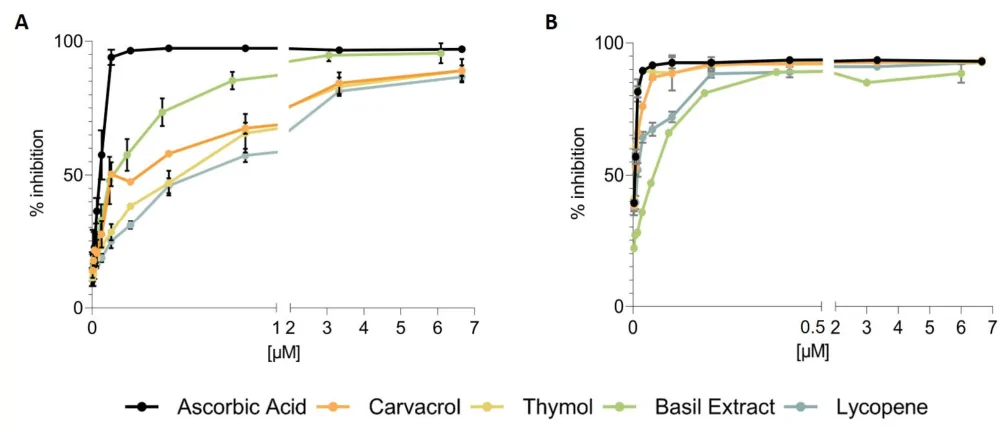
Antioxidant activity of carvacrol, thymol, basil essential oil, and lycopene determined by DPPH (**A**) and ABTS (**B**) radical scavenging assays. Results are expressed as percentage inhibition at increasing concentrations. Ascorbic acid served as the positive control. Data are presented as mean ± SD of three independent experiments.

**Figure 2:**
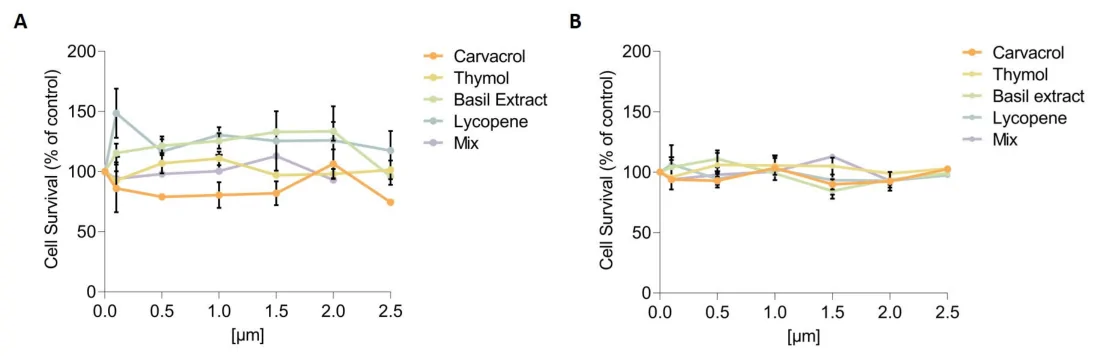
Effects of carvacrol, thymol, basil essential oil, lycopene, and their mixture (2 µM total concentration) on HepG2 cell viability after 24 h (**A**) and 48 h (**B**). Cell viability was determined by MTT assay and expressed as a percentage of untreated cells. Data are presented as mean ± SD of three independent experiments.

**Figure 3:**
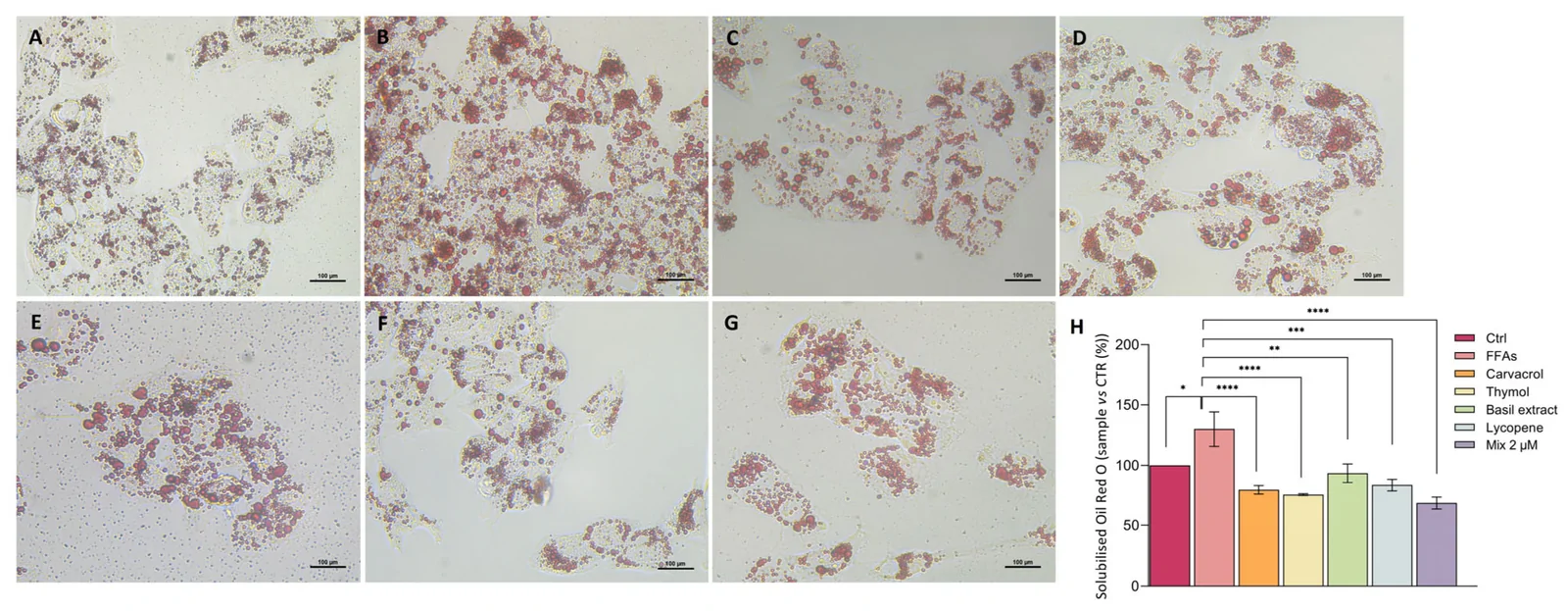
Oil Red O staining of steatotic HepG2 cells after 24 h treatment with carvacrol, thymol, basil essential oil, lycopene, or their mixture (2 µM total concentration). Representative bright-field images (20×): (**A**) control; (**B**) FFAs; (**C**) carvacrol; (**D**) thymol; (**E**) basil essential oil; (**F**) lycopene; (**G**) mixture. (**H**) Quantification of Oil Red O staining, expressed as a percentage of untreated cells. Data are presented as mean ± SD of three independent experiments. * *p* ≤ 0.05, ** *p* ≤ 0.005, *** *p* ≤ 0.001, **** *p* ≤ 0.0001 (two-tailed *t*-test). Horizontal lines indicate statistically compared groups.

**Figure 4:**
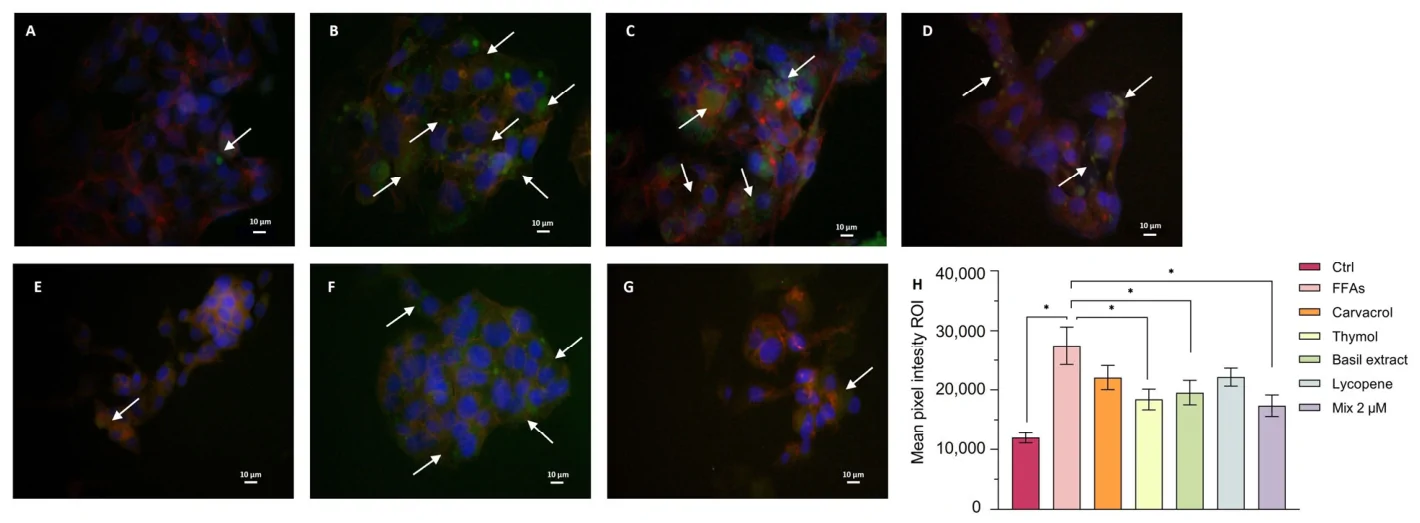
Immunofluorescence staining of steatotic HepG2 cells after 24 h treatment with carvacrol, thymol, basil essential oil, lycopene, or their mixture (2 µM total concentration). Nuclei were stained with DAPI (blue), F-actin with TRITC-phalloidin (red), and lipid droplets with LipidSpot™ (green). Representative images (40×): (**A**) untreated cells; (**B**) FFAs; (**C**) carvacrol; (**D**) thymol; (**E**) basil essential oil; (**F**) lycopene; (**G**) mixture. White arrows indicate lipid droplets. (**H**) Quantification of lipid droplet fluorescence intensity presented as mean ± SD of three independent experiments. * *p* ≤ 0.05 (two-tailed *t*-test). Horizontal lines indicate statistically compared groups.

**Figure 5:**
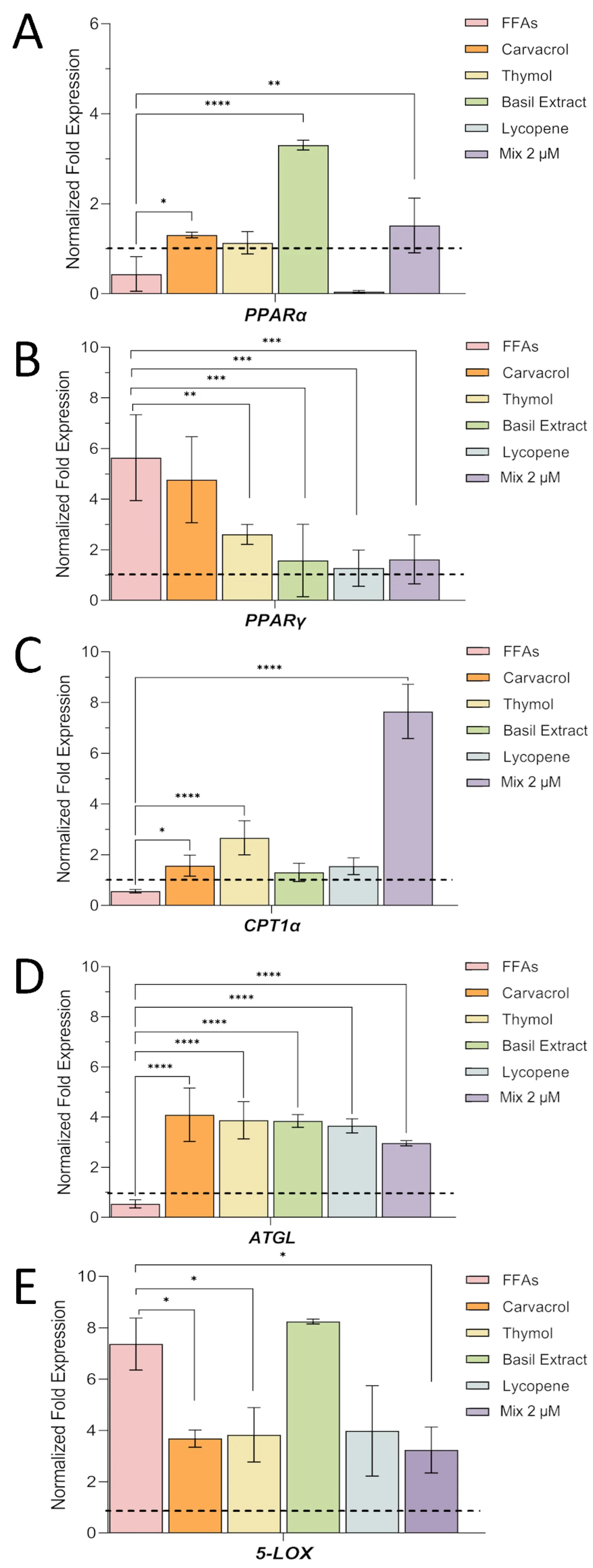
Relative gene expression in steatotic HepG2 cells after 24 h treatment with carvacrol, thymol, basil essential oil, lycopene, or their mixture (2 µM total concentration). Relative mRNA levels of (**A**) PPARα, (**B**) PPARγ, (**C**) CPT1α, (**D**) ATGL, and (**E**) 5-LOX. Data are presented as mean ± SD of three independent experiments. * *p* ≤ 0.05, ** *p* ≤ 0.005, *** *p* ≤ 0.001, **** *p* ≤ 0.0001 (one-way ANOVA followed by Bonferroni’s post hoc test). Horizontal lines indicate statistically compared groups.

**Figure 6:**
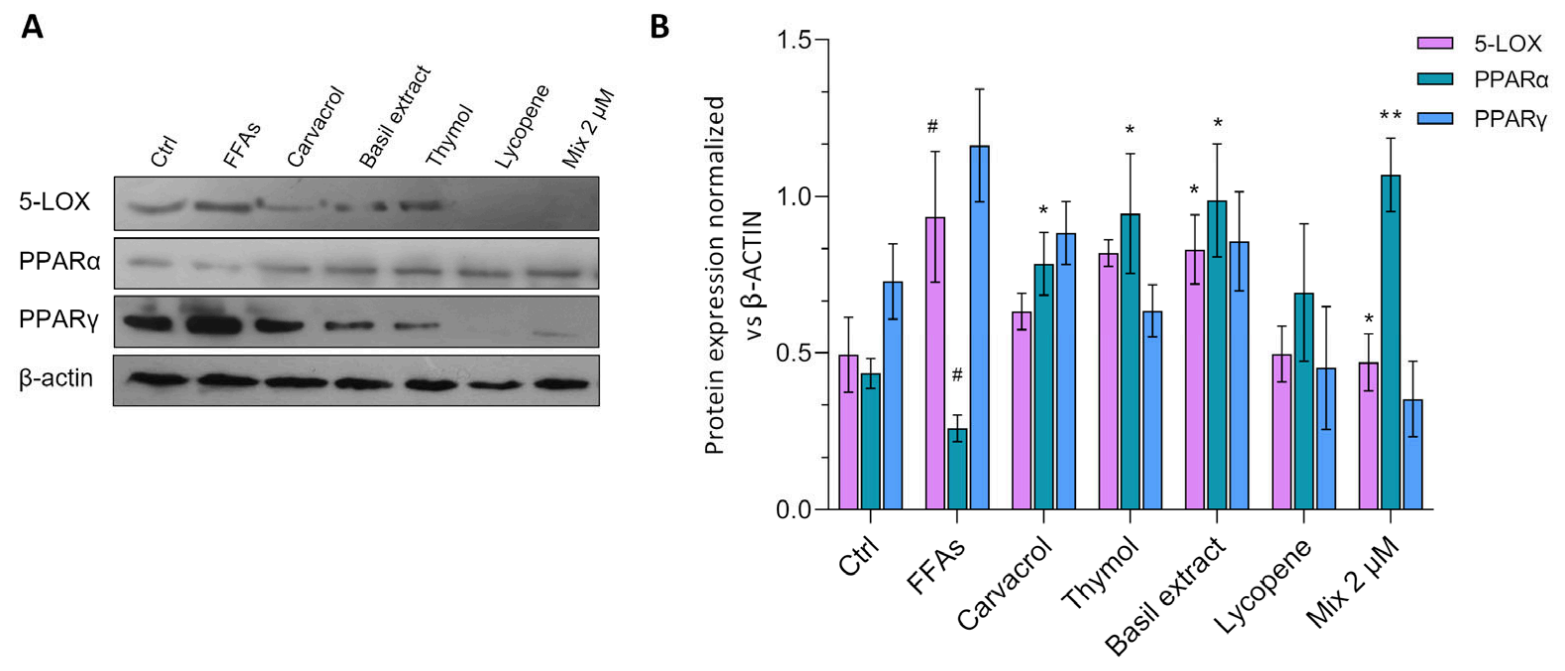
Western blot analysis of protein expression in steatotic HepG2 cells after 24 h treatment with carvacrol, thymol, basil essential oil, lycopene, or their mixture (2 µM total concentration). (**A**) Representative immunoblots of 5-LOX, PPARα, and PPARγ. (**B**) Densitometric analysis normalized to β-actin. Data are presented as mean ± SD of three independent experiments. * *p* ≤ 0.05, ** *p* ≤ 0.005 vs. untreated cells; ^#^ *p* ≤ 0.05 vs. FFAs (two-tailed *t*-test).

**Figure 7:**
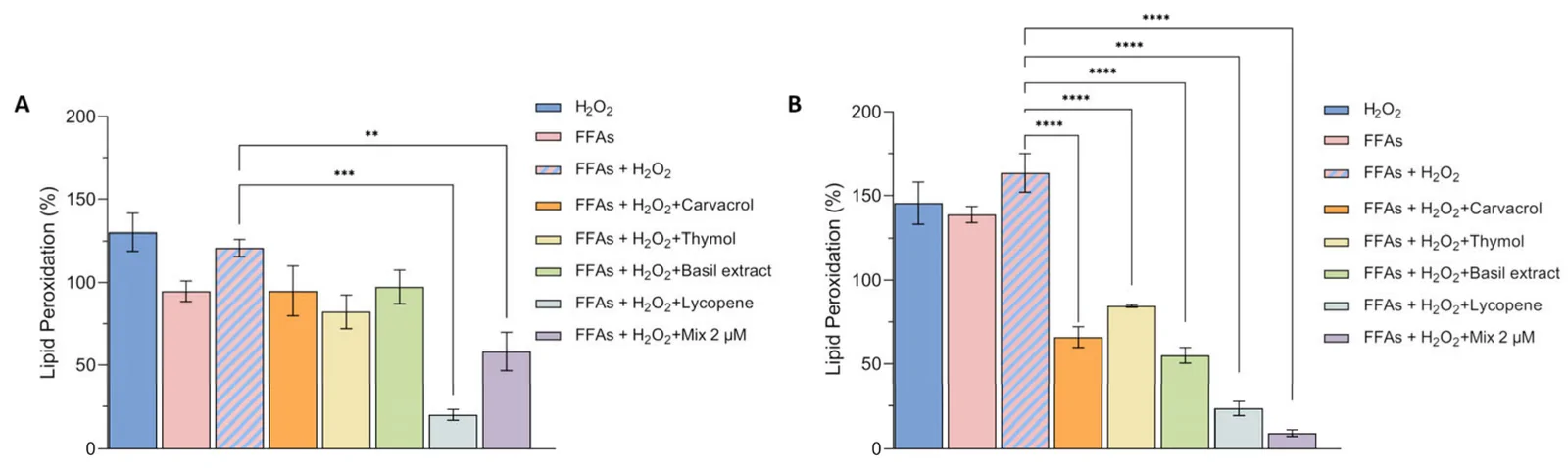
Assessment of lipid peroxidation by TBARS assay in steatotic HepG2 cells treated with natural compounds. (**A**) Preventive setting: cells were treated with carvacrol, thymol, basil essential oil, lycopene, or their mixture before H_2_O_2_ exposure. (**B**) Recovery setting: cells were treated with the same compounds after H_2_O_2_ exposure. Lipid peroxidation levels are expressed as percentage reduction relative to FFAs + H_2_O_2_-treated cells. Data are presented as mean ± SD of three independent experiments, ** *p* ≤ 0.005, *** *p* ≤ 0.001, **** *p* ≤ 0.0001 (two-tailed *t*-test). Horizontal lines indicate statistically compared groups.

**Figure 8:**
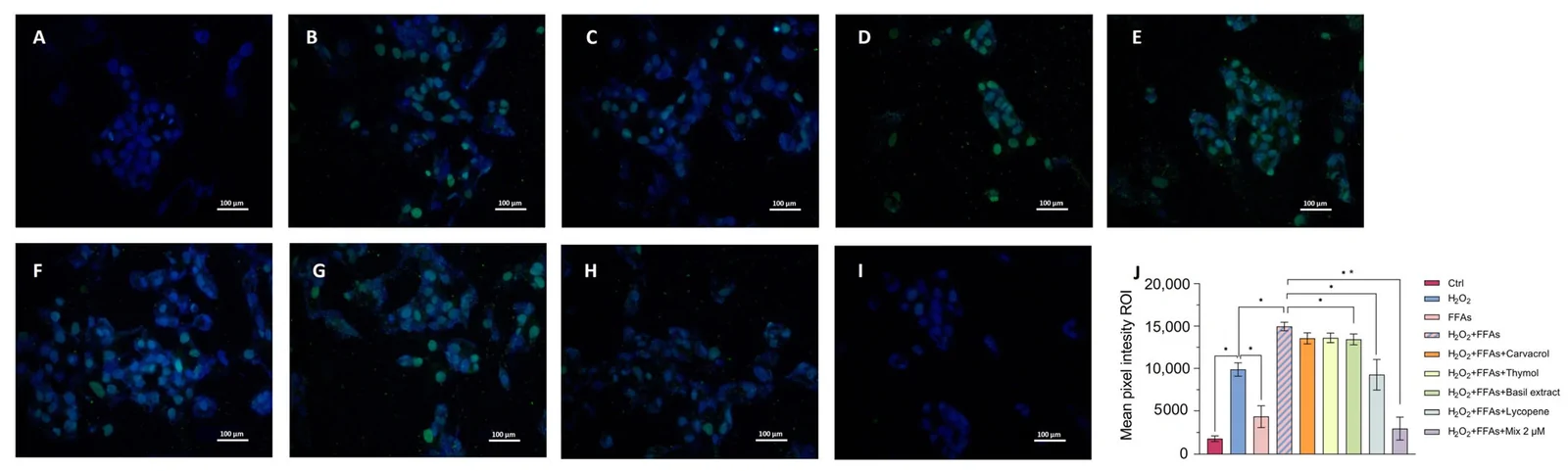
Immunofluorescence detection of 8-oxo-dG in HepG2 cells under oxidative stress conditions and after treatment with natural compounds. Nuclei were stained with DAPI (blue), and 8-oxo-dG was detected in green. Representative images were acquired at 20× magnification: (**A**) control; (**B**) FFAs; (**C**) H_2_O_2_; (**D**) H_2_O_2_ + FFAs; (**E**) H_2_O_2_ + FFAs + carvacrol; (**F**) H_2_O_2_ + FFAs + thymol; (**G**) H_2_O_2_ + FFAs + basil essential oil; (**H**) H_2_O_2_ + FFAs + lycopene; (**I**) H_2_O_2_ + FFAs + mixture. (**J**) Quantification of 8-oxo-dG-positive cells. Data are presented as mean ± SD. * *p* ≤ 0.05, ** *p* ≤ 0.005 (two-tailed *t*-test). Horizontal lines indicate statistically compared groups.

**Table 1:** Primer sequences used for qRT-PCR.

Gene	Forward	Reverse	Amplicon
*PPARα*	5′-ATGGTGGACACGGAAAGCC-3′	5′-CGATGGATTGCGAAATCTCTTGG-3′	124 bp
*PPARγ*	5′- GGGATCAGCTCCGTGGATCT-3′	5′- TGCACTTTGGTACTCTTGAAGTT-3′	186 bp
*CPT1α*	5′-TCCAGTTGGCTTATCGTGGTG-3′	5′-TCCAGAGTCCGATTGATTTTTGC-3′	98 bp
*ATGL*	5′-GTGTCAGACGGCGAGAATG-3′	5′-TGGAGGGAGGGAGGGATG-3′	119 bp
*5-LOX*	5′-CGCCGACTTTGAGAAAATCT-3′	5′-GGCTGCACTCTACCATCTCC-3′	185 bp
*GAPDH*	5′-TGCACCACCAACTGCTTAGC-3′	5′-GGCATGGACTGTGGTCATGAG-3′	87 bp

## Data Availability

All raw data are available upon reasonable request from the corresponding author.

## Supplementary Materials

The following supporting information can be downloaded at https://www.mdpi.com/article/10.3390/molecules31193470/s1. Original Western blot images and Figure S1: Analytical characterization.

## Abbreviations

The following abbreviations are used in this manuscript:

8-oxo-dG	8-oxo-7,8-dihydroguanine
ABTS	2,2′-azino-bis (3-ethylbenzothiazoline-6-sulfonic acid)
ANOVA	analysis of variance
ATGL	adipose triglyceride lipase
5-LOX	5-lipoxygenase
BSA	bovine serum albumin
CPT1α	carnitine palmitoyltransferase 1A
DAPI	4′,6-diamidino-2-phenylindole
DMEM	Dulbecco’s modified Eagle’s medium
DPPH	2,2-diphenyl-1-picrylhydrazyl
ECL	enhanced chemiluminescence
EDTA	ethylenediaminetetraacetic acid
EOs	essential oils
FAAs	free fatty acids
GAPDH	glyceraldehyde-3-phosphate dehydrogenase
H_2_O_2_	hydrogen peroxide
MTT	3-(4,5-dimethylthiazol-2-yl)-2,5-diphenyltetrazolium bromide
NAFLD	non-alcoholic fatty liver disease
NASH	non-alcoholic steatohepatitis
PBS	phosphate-buffered saline
PPARα	peroxisome proliferator-activated receptor alpha
PPARγ	peroxisome proliferator-activated receptor gamma
qRT-PCR	quantitative real-time polymerase chain reaction
RIPA	radioimmunoprecipitation assay buffer
ROS	reactive oxygen species
SDS-PAGE	sodium dodecyl sulphate–polyacrylamide gel electrophoresis
TBARS	thiobarbituric acid reactive substances
TBST	Tris-buffered saline with Tween-20
UPLC	ultra-performance liquid chromatography
