# Supplementary material for: Bioactive Ingredients in the Mediterranean Diet for Hepatic Health: In Vitro Evaluation of Selected Extracts Against Non-Alcoholic Steatosis
Source: Molecules. 2026 Sep 29;31(19):3470. doi: 10.3390/molecules31193470 (PMC13666745; doi:10.3390/molecules31193470)

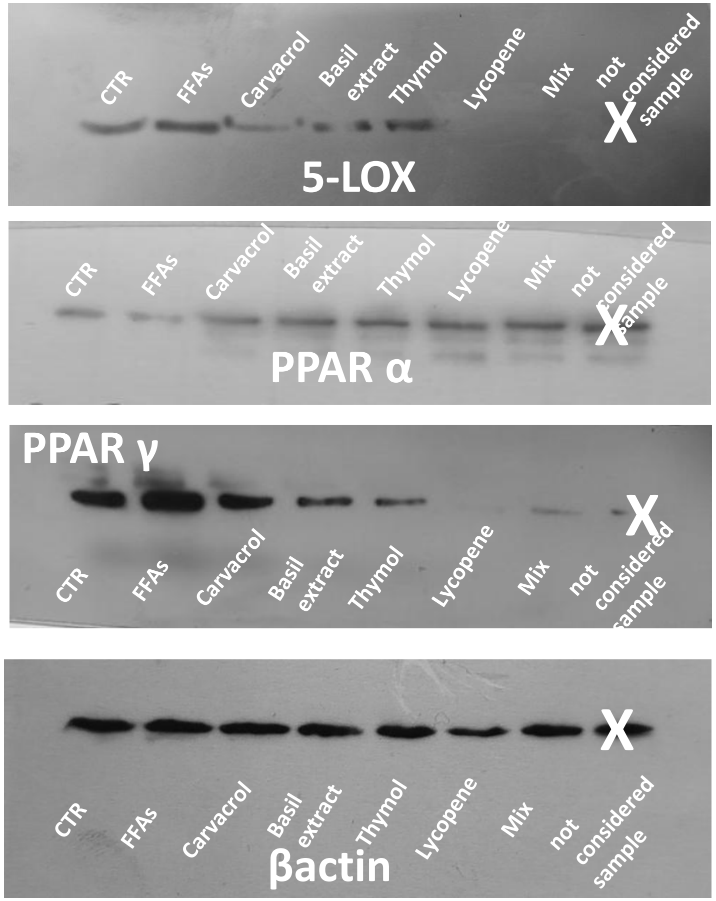
CTR
FFAs
Carvacrol
Basil extract
Thymol
Lycopene
Mix
not considered sample
X
5-LOX
PPAR α
PPAR γ
βactin

In this study, we tested the effect of two mixtures at different concentrations. We decided not to include the second one (the last sample loaded onto the gel) in the manuscript.

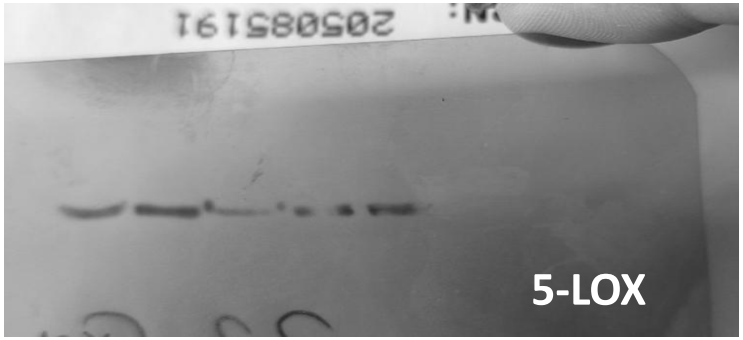

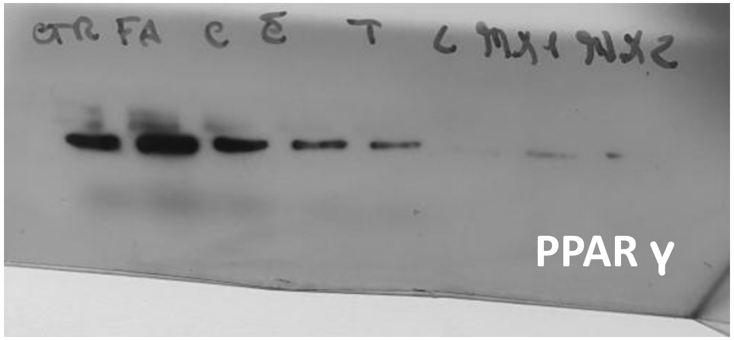

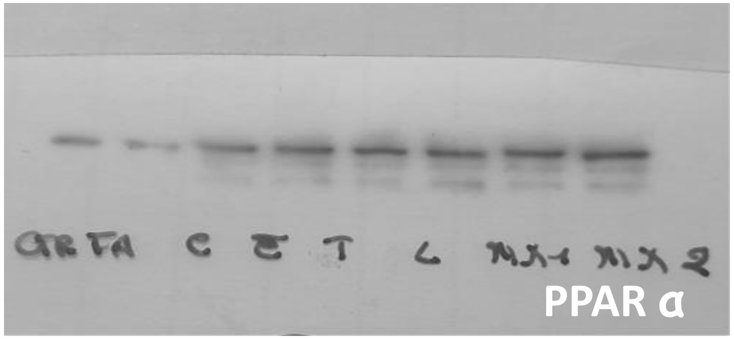

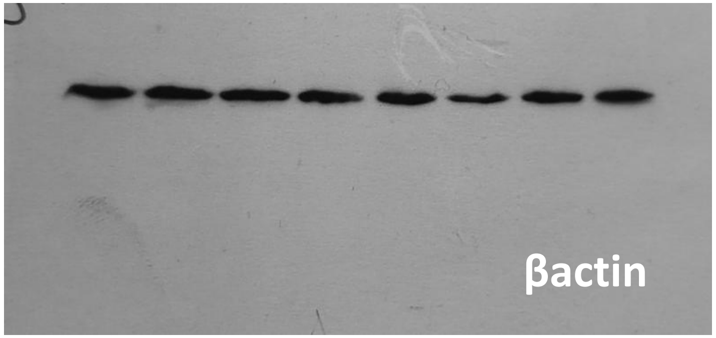

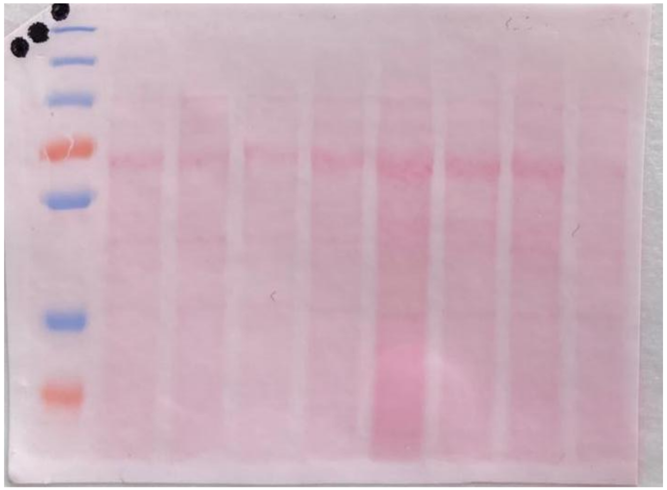

Supplement: Supplementary file 1 [file molecules-31-03470-s001.zip › molecules-4502796-supplementary.pdf]
